# Organ crosstalk: brain-lung interaction

**DOI:** 10.3389/fmed.2025.1655813

**Published:** 2025-08-26

**Authors:** Luciana Mascia, Rosanna D’Albo, Irene Cavalli, Luca Giaccari, Maria Della Giovampaola, Beatrice Donati

**Affiliations:** ^1^Department of Experimental Medicine (DIMES), Campus Ecotekne, University of Salento, Lecce, Italy; ^2^Department of Medical and Surgical Sciences (DIMEC), Alma Mater Studiorum University of Bologna, Bologna, Italy; ^3^Anesthesiology and General Intensive Care Unit, IRCCS Azienda Ospedaliero-Universitaria di Bologna, Bologna, Italy; ^4^Anestesia e Rianimazione, Ospedale Vito Fazzi, Lecce, Italy; ^5^Dipartimento della Donna, del Bambino e di Chirurgia Generale e Specialistica, Università della Campania “L. Vanvitelli”, Naples, Italy; ^6^Department of Anesthesiology, University Medical Center Göttingen, Göttingen, Germany

**Keywords:** acute brain injury, ARDS, brain-lung cross talk, ventilator associated brain injury, mechanical ventilation, neurogenic pulmonary edema

## Abstract

The interaction between the brain and the lungs is bidirectional: ICU patients with acute brain injury develop pulmonary complications, while ARDS patients frequently manifest neurological sequelae. Research is indeed focusing on both aspects of this cross-talk. On one side, ARDS survivors experience poor neurological outcomes both in the short and long term, with high incidence of delirium and post- discharge neurocognitive impairment. The underlying mechanisms have been investigated either in the pre-clinical and in the clinical field. Ventilator associated brain injury is the new recent term used to indicate the brain damage consequent to mechanical ventilation and leading to neuroinflammation and increased brain cells apoptosis. Moreover, prolonged hypoxia, deep sedation, loss of cerebral autoregulation and complications from vv-ECMO during ARDS are potentially sources of brain damage. On the other side, pulmonary complications in patients with acute brain injury follow a double-hit model, recently implemented in a triple-hit hypothesis. According to this theory, the primary brain injury leads to sympathetic hyperactivity, with inflammation and oxidative stress. Thus, the lungs become more vulnerable to develop complications such as neurogenic pulmonary edema and pneumonia. Finally, immune dysregulation and microbiome alterations due to brain-lung cross-talk lead to the worsening of lung injury. In this context, mechanical ventilation strategies aiming to guarantee adequate gas exchange and brain oxygen delivery are essential to prevent this phenomenon cascade. This review purpose is to examine the mechanisms behind brain-lung cross talk, starting from pathophysiological mechanisms, in order to suggest potential new research and therapeutic approaches.

## 1 Introduction

Patients admitted to the intensive care unit (ICU) with acute brain injury often experience extracranial complications, with pulmonary complications being among the most prevalent. These complications not only occur frequently but also serve as independent predictor of poor clinical outcome (1). Conversely, ICU patients suffering from acute respiratory distress syndrome (ARDS) may develop neurological complications, which can significantly impair their prognosis, particularly in the long term (2).

**FIGURE 1:**
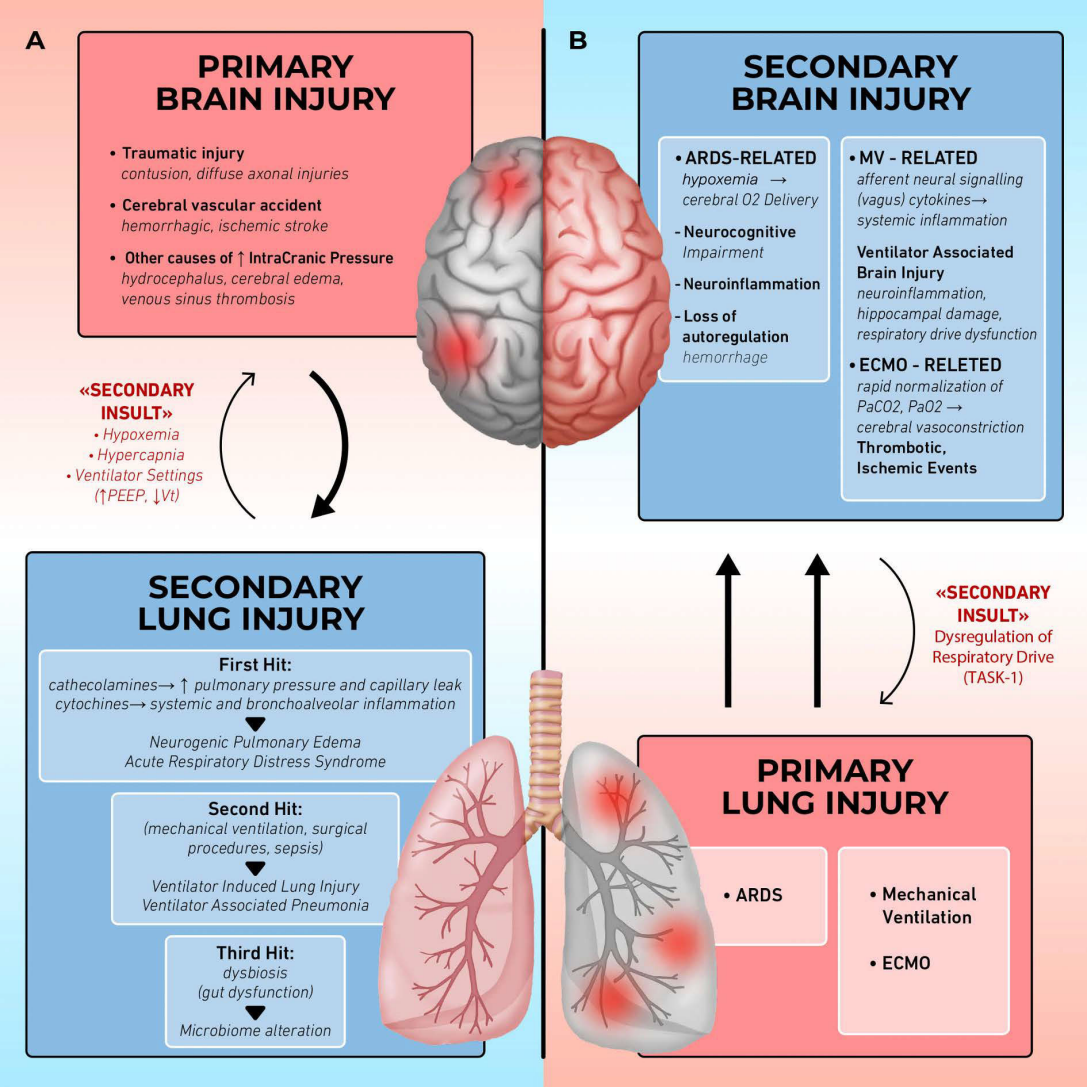
Organ cross talk: lung brain interaction. **(A)** Brain injury induces secondary lung injury: in patients with acute brain injury the release of catecholamines and cytokines alters pulmonary barrier, leading to complications such as neurogenic pulmonary edema (NPE) and acute respiratory distress syndrome (ARDS), further complicated by ventilator-induced lung injury (VILI) and ventilator associated pneumonia (VAP). Hypoxemia, hypercapnia, and ventilatory setting may further impair brain function (secondary insults). **(B)** Lung injury induces secondary brain injury: in patients with acute respiratory failure, the occurrence of poor oxygenation, the application of mechanical ventilation and extracorporeal membrane oxygenation (ECMO) may cause neurocognitive impairment, ventilator-associated brain injury (VABI), thrombo-ischemic events. Respiratory drive impairment may further affect the lung function (secondary insults).

The relationship between the brain and lungs is therefore bidirectional: acute neurological injury can lead to pulmonary complications (3–5), while lung injury, including ventilator-induced lung injury (VILI), can disrupt brain homeostasis (Figure 1) (6, 7). This review explores the dynamic interplay between the brain and lungs, highlighting the mutual impact of acute damage in one organ system on the other.

## 2 Primary lung injury and secondary brain injury

The cornerstone of effective management for patients with Acute Respiratory Distress Syndrome (ARDS) remains protective mechanical ventilation (8). This approach plays a critical role in supporting respiratory function across diverse clinical scenarios, ranging from acute lung injury to providing respiratory assistance during organ donation procedures (8, 9). By mitigating the risk of ventilator-induced lung injury (VILI), protective ventilation minimizes additional harm to already compromised lung tissue (10, 11).

Recent epidemiological data highlight the significant burden of ARDS in critical care settings. A comprehensive report on its incidence revealed that ARDS accounts for 10% of all intensive care unit (ICU) admissions and is present in more than 20% of patients requiring mechanical ventilation. The prognosis remains concerning, with an ICU mortality rate of approximately 35%, which escalates to 40% when considering overall in-hospital mortality (12).

For survivors of ARDS, the challenge extends far beyond the ICU. Long-term studies indicate that many patients experience persistent reductions in functional capacity, even at 1-year follow-up (13). Neurological outcomes are particularly alarming: almost 50% of ARDS survivors face enduring neurocognitive impairments 2 years after ICU discharge, accompanied by a diminished quality of life (2).

The neurological sequelae of ARDS are not merely functional but may also stem from organic damage to the central nervous system (CNS). A recent review underscores the gravity of this issue, reporting an incidence of acquired brain injury or poor neurological outcome in almost 80% of ARDS patients (6). However, the variability in study methodologies and the heterogeneity of patient populations have contributed to these wide-ranging estimates.

While the pulmonary repercussions following brain injuries are well documented, less is known about the reverse relationship, how primary lung diseases, such as ARDS, contribute to secondary brain damage (Figure 1).

Among the neurological manifestations, delirium stands out as a frequent and significant complication. Studies reveal that ICU patients undergoing mechanical ventilation experience delirium in 60% to 80% of cases (14). More specifically, prolonged mechanical ventilation (greater than 12 h) is associated with even higher rates of delirium, affecting 70%–80% of ventilated patients compared to 20%–40% of non-ventilated ICU patients (15). This delirium not only complicates clinical management but also has long-lasting implications for cognitive recovery and overall well-being (2).

### 2.1 Ventilator associated brain injury

In this context, the concept of ventilator associated brain injury (VABI) is gaining ground in the research field (16). VABI is defined as a brain damage consequent to mechanical ventilation in patients without primary brain injury. It is important to highlight that the definition of VABI requires the presence of mechanical ventilation and the absence of confounders for the evaluation of the brain functions (i.e., sedation, hyperthermia, electrolytes abnormalities, hypoxia etc.,).

#### 2.1.1 Pathophysiological mechanisms

There is already strong evidence, both from animals and humans studies, of the presence of VABI. Preclinical studies on mice and pigs have shown that mechanical ventilation promotes neuroinflammation and neuronal apoptosis in a dose-dependent manner. Particularly, the higher the tidal volume, the driving pressure and the mechanical power, the more relevant and numerous the brain lesions (17). Moreover, a cognitive impairment (18) very similar to Alzheimer’s disease (19) is proportional to the duration of mechanical ventilation.

The underlying mechanism causing neuroinflammation and neuronal apoptosis is thought to be due to the continuous stretching of lung fibers due to positive pressure ventilation. One study showed that mechanical ventilation causes the increased pulmonary toll-like receptor-4 expression, which initiates the inflammatory cascade (20, 21). Lung stress and strain promote in this way the neuronal hyperactivity, whit production of inflammatory cytokines and inhibition of pro-survival pathways. The apoptosis signaling is, at the same time, triggered by the vagus afferences in turn stimulated by the cyclic alveolar stretch and by the inflammatory mediators present in the lung environment (21). In particular, the vagal signaling seems to lead to an hyperdopaminergic state in the hippocampal region (22). The results are an augmentation of brain inflammation and an increase in neuronal apoptosis, both leading to brain injury. Several markers such as the c-fos gene (17) or the elevated presence of microglia, even in pigs without previous lung injury (23), have been identified to detect neuronal activity and inflammation, while the presence of apoptosis has been highlighted by terminal deoxynucleotidyl transferase dUTP nick end labeling (TUNEL) positive cells, by phosphorylation of glycogen synthetase kinase 3b (GSK3b) or by cleavage of poly-adenosine-diphosphate-ribose polymerase-1 (PARP-1) (24).

Finally, in an animal model it has been shown that mechanical ventilation reduces the expression of potassium channels TASK-1, involved in the regulation of the respiratory rhythm and drive. The higher the tidal volume, the lower was the expression of this channel (25).

#### 2.1.2 Clinical studies

Hypothesis from the pre-clinical field have been confirmed by clinical observational studies evaluating the role of mechanical ventilation on neuronal and long-term cognitive outcome in humans. In this perspective, the presence of delirium in mechanically ventilated patients has been largely investigated (24). However, these patients are often critically ill, always sedated and physically inactive, so that it is difficult to show a direct link between mechanical ventilation and delirium. Plus, studies evaluating the prevalence of delirium identified several risk factors for its developing. Moreover, it is hard to eliminate the effects of confounding factors, particularly sedation, which in most cases allows a better ventilation strategy. Despite this, in several studies mechanical ventilation has been identified as an independent variable associated with delirium and, vice versa, delirium has been shown to be independently associated with prolonged need of respiratory support. Also, there was a higher likelihood of developing delirium in mechanically ventilated patients than in not ventilated ones. Of note, this does not means a causal link between mechanical ventilation and the occurrence of delirium, but rather a correlation between them (24). Consequently delirium, in turn, could be a predictor of greater likelihood for chronic cognitive impairment after critical illness (24). A prospective multicenter study involving general ICUs patients found that longer duration of delirium during the ICU stay was related with worse global cognition after discharge. This type of cognitive impairment shares some similarities with Alzheimer’s disease but affects a broader range of cognitive domains; in fact, its characteristics closely resemble those seen in moderate traumatic brain injury (26).

Nevertheless in the clinical scenario, the main research focus has been on ARDS definition and on the consequent need of the optimal ventilatory strategy. However, the impact of ARDS and, in general, of critical illness on the quality of life is now well recognized. In a pivotal large prospective study (13) on ARDS patients, a global functional limitation 1 year after discharge from ICU was demonstrated, but still the post-discharge cognitive impairment was not clearly identified (27). The hypothesized mechanisms underlying the neurological sequalae after ARDS were the prolonged hypoxia, the effect of systemic inflammatory cytokines, the use of sedative drugs and the possible presence of emboli in the CNS (27).

#### 2.1.3 Potential therapeutic target

Given the early stage of research in this field, there is currently no general consensus for managing VABI. Nonetheless, based on our understanding of its underlying pathophysiology, several strategies have been proposed to mitigate the damage caused by mechanical ventilation. First, reducing lung stress and strain with protective ventilatory strategies, which gently impact on the lungs, is the most straightforward choice in mechanically ventilated patients (28). In addition, approaches leading to reduction in mechanical ventilation days (e.g., resolution of the primary cause, optimal sedation strategy, improvement in breathing coordination) and therapies for the delirium itself (e.g., alpha-2 agonists) may have a positive impact in reducing post-discharge neurological sequalae (29).

Besides it has been hypothesized that a negative pressure ventilation, obtained by diaphragm stimulation, could reduce brain damage. Negative pressure ventilation has been proposed as more physiological because it is thought to exert less stimulation on lung mechanoceptors, thus reducing lung stress. Indeed, Bassi et al. found that pigs receiving transvenous diaphragmatic stimulation showed lower hippocampal apoptotic indices and reactive astrocytes compared to mechanically ventilated ones, both in not injured lungs and in ARDS models (30–32). They also showed that in the ARDS model, the diaphragmatic stimulated group had a reduced mechanical power (33). The investigators concluded that diaphragmatic stimulation could have a neuroprotective action. An additional point in favor of negative pressure ventilation could lie in a lower hemodynamic impact, which helps cerebral perfusion (34).

Another potential approach derives from neurological studies about the default mode network and the connection across multiple brain areas. It has been showed that olfactory bulb is largely involved in promoting brain cells interaction, being stimulated by nasal airflow (35). Since the airflow is deviated from the nose during mechanical ventilation, this could lead to impaired cognitive function. In fact, the same research group hypothesized that the absence of nasal airflow during mechanical ventilation could be a possible mechanism of impaired brain activity. They found that non-invasive stimulation of olfactory bulb by nasal air puffs during mechanical ventilation resulted in better coherence and synchrony between brain regions, mostly in the DMN (36). This was further confirmed by another study in which working memory performance in rats was significantly higher in the air-puff group compared to control (37). Consequently, olfactory bulb stimulation and olfactory epithelium electrical stimulation (OEES) may offer an alternative therapeutic target. In fact, a clinical study demonstrated that such stimulation activated the default mode network in comatose patients following an opioid overdose (40204831).

Finally, a new research line is the pharmachological approach such as mechanoceptor and inflammatory pathway blockade (21).

### 2.2 Brain injury related to cerebral autoregulation impairment

Another potential mechanism of brain injury during primary lung damage is not properly related to mechanical ventilation, but instead to loss of cerebral autoregulation in the context of pulmonary diseases. This has been hypothesized primarily in preterm infants affected by respiratory distress syndrome due to lack of surfactant. Indeed, RDS is a well-established risk factor for peri- and intraventricular hemorrhage in these patients. Additionally, there is evidence suggesting that repeated episodes of impaired cerebral autoregulation may contribute to brain damage (38, 39).

Finally, extracorporeal support may serve as a last resort for patients with refractory hypoxemia. However, in patients receiving venovenous-ECMO, cerebral complications–both hemorrhagic and thrombotic–are common. These events are primarily influenced by pre-cannulation conditions, patient age, anticoagulation therapies, and the duration of ECMO support.

In this context, rapid normalization of PaCO2 can result in cerebral vasoconstriction leading to a decrease in cerebral oxygenation eventually predisposing to cerebral infarction and intracerebral hemorrhage (40–42).

## 3 Primary brain injury and secondary lung injury

Patients with acute brain injury are at increased risk of developing extracranial complications that may have a detrimental impact on outcome (43, 44). The importance of lung injury as a potential extracranial complication in patients with acute brain injury stems from its ability to independently predict unfavorable outcomes (5, 44–46). Observational studies have consistently shown that mortality is higher in patients with both brain and lung injuries compared to those with brain injury alone (1, 44).

Early descriptions of lung damage in the context of acute brain injury ranged from 22% to 30%, with a higher incidence observed in patients sustaining head injuries compared to those experiencing other forms of neurological damage such as subarachnoid hemorrhage (5, 44, 45).

However, over the years, a lower incidence of lung damage during head injury has been observed, now estimated around 20% (46). This discrepancy is likely attributable to the stricter diagnostic criteria for acute respiratory distress syndrome (ARDS), in comparison to previous standards.

Significant pathophysiological differences exist among the major forms of acute brain injury–namely traumatic brain injury (TBI), aneurysmal subarachnoid hemorrhage (aSAH), intracerebral hemorrhage (ICH), and central nervous system (CNS) infections–which critically influence the nature and severity of secondary pulmonary manifestations within the framework of brain–lung crosstalk.

In TBI, intracranial pressure (ICP) elevation is typically acute and driven by mass lesions (e.g., hematomas), vasogenic edema, or diffuse axonal injury. These patients frequently sustain concomitant thoracic trauma (in up to 30%–40% of cases), which complicates oxygenation and increases the risk of acute respiratory distress syndrome (ARDS) (1, 43). Furthermore, systemic inflammatory responses and catecholamine surges can exacerbate pulmonary dysfunction (42, 46).

In aSAH, ICP fluctuations may result from acute hydrocephalus or delayed cerebral vasospasm. Patients with aSAH commonly have pre-existing pulmonary comorbidities, such as chronic obstructive pulmonary disease (COPD), often related to smoking–a recognized risk factor for aneurysmal rupture (5, 44). Post-ictal sympathetic hyperactivity frequently induces neurogenic pulmonary edema (NPE) and transient cardiopulmonary dysfunction (3).

In ICH, parenchymal hematoma expansion and vascular disruption rapidly compromise cerebral perfusion and often coincide with labile systemic blood pressures. Acute hypertensive episodes may promote pulmonary edema or trigger stress-induced cardiomyopathy (e.g., Takotsubo syndrome), thereby predisposing to respiratory complications (47).

Finally, CNS infections–including encephalitis and bacterial meningitis–are frequently associated with systemic sepsis. The resulting systemic inflammatory response can impair cerebral autoregulation and compromise pulmonary function, significantly elevating the risk of ARDS and ventilator-associated pneumonia (VAP) (48, 49).

### 3.1 Pathophysiological mechanisms

The hypothesis of brain-lung crosstalk is based on a *double hit model* (50). The activation of inflammatory mediators and the release of catecholamines predisposes to a systemic immune response and the activation of different molecular pathways. At the pulmonary level, the increase in hydrostatic pressure within the vessels leads to increased capillary permeability and capillary vasoconstriction, triggering endothelial dysfunction and molecular infiltrates.

Recently, this model has been further expanded in the *triple hit hypothesis* (51). The initial brain injury triggers sympathetic hyperactivity, leading to inflammation and oxidative stress (*first hit*). The lungs become more vulnerable to secondary procedures such as mechanical ventilation (*second hit*). Finally, dysbiosis and gut dysfunction in brain injury patients initiate a cascade of events with immune dysregulation and microbiome alterations, which also affect lung tissue (*third hit*). All of this leads to the development or worsening of lung injury (Figure 1).

The first hit, known as neurogenic pulmonary edema (NPE), was first described by Theodore and colleagues several decades ago (52). Subsequently, this phenomenon has been documented in clinical settings through observational studies, wherein pulmonary edema unrelated to cardiogenic causes was observed in patients with acute brain injury, with a biphasic incidence occurring between the second and fourth days and then between the fifth- and eleventh-days post-injury (3, 4, 53). NPE is defined as a form of respiratory distress related to the presence of acute brain injury and not attributable to heart failure or fluid overload (3). It is due to a sudden increase in intracranial pressure (ICP), resulting in a reduction in cerebral perfusion pressure (CPP) or direct damage to the brainstream and hypothalamus (54). This leads to a dysregulation of catecholamine homeostasis with massive α-adrenergic activation resulting in vasoconstriction and increased blood pressure with a massive blood shift from the systemic to the pulmonary circulation (54). The increase in hydrostatic pressure leads to fluid leakage and endothelial dysfunction. Besides acute brain damage triggers systemic inflammation, leading to the release of proinflammatory cytokines. This, in turn, increases capillary permeability in the pulmonary vessels, resulting in edema (55). The clinical presentation is characteristic of respiratory failure, with chest X-rays revealing bilateral pulmonary infiltrates (54). Symptoms appear 30–60 min after brain injury or within 12–24 h after injury. The first phenotype, the fulminant one, has a higher mortality rate than the second. The severity of the edema is directly related to the severity of the acute brain injury; in 50% of cases, resolution occurs within 72 h of onset. Intracranial hypertension increases the levels of extravascular lung water in poorly aerated lung areas and enhancing lung inflammation (56).

The systemic inflammatory response initiated by the primary brain injury increases the risk of infections and, more specifically, pneumonia (16). All these mechanisms contribute to the development of ALI/ARDS in patients with acute brain injury.

Finally, brain-lung microbiome interactions are gaining increasing importance. All factors (e.g., oxygen tension, blood pH, temperature) that cause microbial dysbiosis of the respiratory tract change the status of the alveoli contributing to the development of complications, from ventilator associated pneumonia (VAP) to organ failure (57). Specifically, the increased incidence of VAP between 21% and 60%, is due to several factors. First, altered level of consciousness and aspiration are recognized as risk factors (58). Second the need for prolonged mechanical ventilation and sedation (59). Third, dysphagia associated with brain injury is associated with a higher incidence of pneumonia (59). Finally, the systemic inflammatory response can lead to the development of nosocomial pneumonia (59).

### 3.2 Acute brain injury and mechanical ventilation strategies

Tracheal intubation and MV are the most frequent artificial supports in patients with ABI, since this condition is often associated with the impairment of airways patency and of respiratory drive. Moreover, MV plays an important role, especially during the first days after ABI, in controlling two fundamental variables related to the pathophysiology of brain injury: oxygenation and carbon dioxide tension.

The arterial content of oxygen (CaO_2_), together with cerebral blood flow (CBF), are the essential elements to provide an adequate delivery of oxygen to the brain; the importance of this mechanism relies on a peculiar characteristic of the brain tissue, that is unable to store energy and O_2_ despite its high metabolic rate. An alteration in CaO_2_ may therefore impair cerebral aerobic metabolism, worsening the acute brain damage (60, 61).

On the other hand, hypercapnia is a well-known factor that contributes to the worsening of ABI. PaCO_2_, altering extravascular pH, is the strongest factor that controls the tone of cerebral vessels and consequently cerebral blood flow (CBF) (62). Hypercapnia, associated with acidosis, determinates cerebral vasodilation, with an increase in brain volume and intracranial pressure (ICP). Conversely, hypocapnia induces alkalosis, determining cerebral vasoconstriction, that may help in reducing ICP but also cause or worsen cerebral ischemia, especially if prolonged during the time (63).

Mechanical ventilation (MV) is therefore fundamental in ABI patients to maintain and restore brain physiology after brain injury; however, MV itself may interfere with cerebral physiology and worsen brain injury.

In recent decades, the use of ventilation with low tidal volumes (Vt) and moderate to high positive end-expiratory pressure (PEEP), known as protective ventilation, has demonstrated efficacy not only in patients with acute respiratory distress syndrome (ARDS) but also in those requiring mechanical ventilation for extrapulmonary conditions. However, protective ventilation may be challenging in ABI: low Vt may lead to hypercapnia, increasing CBF and ICP, with high PEEP values worsening this effect. Moreover, no evidence has been reached about the optimal targets of PaCO_2_ and PaO_2_ in these patients. However, a consensus conference (64) published by ESICM in 2020 suggests to maintain PAO_2_ between 80 and 120 mmHg in brain injured patients with or without a pathological ICP elevation. Concerning PaCO_2_, the optimal range in patients without intracranial hypertension is 35–45 mmHg; short-term hyperventilation is suggested in case of brain herniation, but no consensus has been reached about hyperventilation in case of intracranial hypertension.

#### 3.2.1 Positive end-expiratory pressure

Positive end-expiratory pressure (PEEP) is used to prevent alveolar collapse during the expiratory phase, improving oxygenation and lung compliance. Consequently, the application of PEEP may have a positive impact in brain tissue oxygenation (PbtO_2_) (65), especially in patients with lower PbtO_2_ at baseline (66). On the other hand, PEEP alters intrathoracic pressure, determining a reduction of venous return, thus reducing cerebral venous outflow, with a worsening effect on CBF and ICP (67). These results are related to the effect of PEEP in terms of alveolar recruitment or overdistension.

#### 3.2.2 Tidal volume

Tidal volume (Vt) multiplied by respiratory rate is equal to V_*min*_. These variables represent the determinant of PaCO_2_ that regulates CBF. For this reason, neurological patients are often hyperventilated to decrease ICP. However, hyperventilation has been demonstrated to be one of the components of ventilator-induced lung injury (VILI). VILI not only manifests its effect on pulmonary parenchyma, but also induce the activation of a systemic inflammation determining secondary insult to the brain (67, 68).

The absence of a standardized protocol to ventilate brain injured patients has been revealed by an international survey conducted by the European Society of Intensive Care Medicine (ESICM) (69), that showed not only the lack of specific protocols in clinical practice for MV and weaning but also a great variability all over the world.

Despite these observations, protective ventilation is still suggested with caution in the setting of neurocritical care for different reasons, like the high prevalence of ARDS in ABI patients (46) and the presence of a neuroinflammatory response to acute brain injury that can predispose to the development of lung injury (67, 68).

In this perspective, ESICM guidelines (64) suggest using a protective ventilation strategy in patients with ABI and ARDS but, at the same time, underly the importance to detect any ICP alteration when increasing PEEP values. On the other hand, the same consensus does not give any recommendation or suggestion for patients with both ABI and ARDS in case of pathological ICP elevation. Moreover, no strategy is suggested in patients with isolated ABI to prevent the occurrence of ARDS or other pulmonary complications.

The lack of evidence about this last statement is confirmed also by a recent meta-analysis (70), that involved 5,639 patients and did not find any improvement in 28 days or in-hospital mortality and in the occurrence of ARDS using a regimen of protective ventilation. These results has been recently confirmed by a multicentre randomized clinical trial (71), that found an increased rate of ARDS, death or ventilator-dependency at 28 days after ABI using protective ventilation rather than conventional ventilation settings to prevent the occurrence of ARDS.

#### 3.2.3 Prone position

Prone position is one of the most important rescue maneuvers for severe ARDS. Unfortunately, due to their peculiar condition, patients with ABI are usually excluded from studies involving this strategy: a recent meta-analysis found that brain injury was an exclusion criterion in 75% of the trials assessing prone positioning in ARDS patients (72). So far, few studies concerning the use of this maneuver in patients with both ABI and ARDS are available. Retrospective analyses found that prone position can be associated with ICP increases, but that the level of increase is higher in patients with higher ICP values at baseline (72, 73) and that this event may not be associated with a reduction in CPP and in CBF (67). At the same time, prone position was found to be effective in terms of improving oxygenation and respiratory system compliance, especially in those group of patients with severe ARDS and higher FiO_2_ and PEEP (74). In conclusion, prone position can be considered in patients with ABI and severe ARDS, considering both the severity of respiratory insufficiency and the severity of intracranial hypertension at baseline.

#### 3.2.4 Extracorporeal support

Extracorporeal membrane oxygenation (ECMO) and extracorporeal CO_2_ removal (ECCO_2_R) are used for ARDS in the most severe cases (75). During protective ventilation, ECCO_2_R allows decarboxylation, lowering PaCO_2_ and avoiding respiratory acidosis, while ECMO controls both oxygenation and carbon dioxide removal.

Both techniques can be useful in ABI patients with ARDS, but their use is still mostly avoided in this context because of the related side effects, especially the increased bleeding risk associated with thrombocytopenia and with heparin bolus and continuous infusion.

However, some case reports (76, 77) suggest that the use of ECMO after TBI might be feasible if associated with strategies that decrease the risk of bleeding, such as a lower activated clotting time (ACT) targets or avoiding anticoagulation.

Concerning extracorporeal decarboxylation, a retrospective study describes the use of pumpless extracorporeal lung assist (pECLA) in patients with severe traumatic brain injury, describing a reduction in PaCO_2_ and in the volume of CSF daily drained associated with a better ICP control (78). All together these data suggest that, even if extracorporeal strategies are difficult to be applied in ABI patients, they may be taken into consideration in selected cases.

## 4 Conclusion

In conclusion the dynamic interplay between the brain and lungs in different clinical conditions may influence the acute damage in one organ system and its influence on the other: acute neurological injury can lead to pulmonary complications, while lung injury, including ventilator-induced lung injury (VILI), can disrupt brain homeostasis. Several therapeutic opportunities are available but require a deep knowledge of pathophysiology and thorough level of monitoring of both the respiratory and cerebral functions.
